# Student-created digital artefacts for health promotion and disease prevention: A scoping review

**DOI:** 10.4102/sajp.v82i1.2298

**Published:** 2026-03-31

**Authors:** Anke van der Merwe, Lizemari Hugo

**Affiliations:** 1 School of Health and Rehabilitation Sciences, Faculty of Health Sciences, University of the Free State, Bloemfontein, South Africa; 2 School of Nursing, Faculty of Health Sciences, University of the Free State, Bloemfontein, South Africa

**Keywords:** clinical education, digital artefacts, disease prevention, health promotion, undergraduate healthcare students

## Abstract

**Background:**

Digital health promotion is critical in addressing global disease burden, yet little is known about how healthcare educators train undergraduate students to create these artefacts for health promotion and disease prevention. The Theory of Planned Behaviour (TPB) framework underpinned the study.

**Objectives:**

To describe the creation of digital artefacts for health promotion and disease prevention by undergraduate students in health professions programmes.

**Method:**

The review used the Johanna Briggs Institute (JBI) Preferred Reporting Items Extension for Scoping Reviews (PRISMA-ScR) guidelines, systematically searching 13 databases with information scientist assistance. Studies describing digital artefacts created by undergraduate healthcare students were included. Data were inductively and thematically analysed.

**Results:**

Of the 314 records, 11 articles from mostly high-income countries focussing on medical and nursing students were included after screening. Digital artefacts included videos, social media content, and multimedia materials targeting populations. Students demonstrated positive attitudes towards creation, influenced by empowerment and knowledge gains, with target populations reporting increased knowledge and cultural relevance. Subjective norms were shaped by institutional support and expert guidance. Students showed perceived behavioural control due to technological competencies. Only two studies reported using educational frameworks.

**Conclusion:**

This study emphasises digital artefacts’ effectiveness in health promotion, highlighting their reach to diverse populations. Findings stress the importance of support in assisting students to create accurate, culturally relevant health messages for the evolving healthcare environment.

**Clinical implications:**

Healthcare curricula must adapt to include digital health promotion skills, requiring educators to update teaching approaches to prepare graduates for modern healthcare delivery.

## Introduction

### Background

The growing global burden of diseases paints a grim picture across healthcare systems (Vollset et al. 2021). A crucial strategy to decrease the burden of disease has been presented through health promotion and disease prevention by encouraging healthy behaviours and reducing risk factors (Kaminsky et al. 2022; Qoseem et al. 2024). To drive this change, the World Health Organization (WHO) recommends educational and social communication activities as essential support strategies to enhance the adoption of health promotion and disease-preventing practices related to both non-communicable and communicable diseases (WHO 2025).

In response, the digital revolution has fundamentally transformed healthcare communication, enabling unprecedented access to health information for billions while simultaneously creating new challenges for information quality, equity, and patient–provider relationships (Qoseem et al. 2024). Global initiatives such as *Healthy People 2030* (Office of Disease Prevention and Health Promotion 2024) emphasise the need to empower individuals and communities through accessible, understandable and actionable health information. Literature describes the use of different materials and formats to disseminate health-related information to communities (Koh et al. 2021). While healthcare providers have traditionally been the primary source of health education using traditional materials, including pamphlets, posters and printed educational content, there’s a need for more accessible solutions to disseminate health information to wider audiences (Koh et al. 2021; Mangadu 2014). In recent years, health information dissemination has been transformed by digital interventions explicitly designed to support disease prevention and health promotion (Abdel-Aziz et al. 2022; Manapurath, Veetil & Kamath 2023). In our study, a *digital artefact* refers to any health communication product designed for health promotion and disease prevention created using technology. These formats include but are not limited to videos, podcasts, blogs (text- or video-based), wikis, animations, and moderated social media content (Koh et al. 2021; Sneed 2016; Walton, Childs & Jugo 2019).

Increasingly, communities across the globe, including resource-constrained settings, have access to mobile phones, tablets, computers, and wearable devices, enabling the creation and sharing of health information across platforms (Lupton 2014; Office of Disease Prevention and Health Promotion 2024; Wagner 2021; WHO 2025). To drive meaningful change in health behaviours using digital health communication, healthcare professionals must be adept at creating and presenting people-centred digital health communication that is relevant to the unique needs of the communities they serve (Abdel-Aziz et al. 2022; Houbby, Abdelwahed & Kumar 2020; Maeda & Socha 2021; Vollset et al. 2021). Consequently, undergraduate healthcare students should graduate with the digital skills to communicate preventive healthcare messages effectively, both to individual patients and to broader communities in various formats. This positions health professions educators as key drivers in equipping students with the ability to design, manage, and evaluate these digital artefacts as part of community health promotion strategies (Del Riccio et al. 2023; Stellefson et al. 2020).

With increasing access to technology, health communication delivered via digital platforms should be strategically designed to maximise reach and impact (Abdel-Aziz et al. 2022; Houbby et al. 2020). Such digital communication should not only inform but also foster knowledge gain, support the adoption of positive health behaviours, and enhance individuals’ self-efficacy (Dehsorkhi et al. 2023). As with traditional health promotion material development, there are systematic steps such as situational analysis, capacity building, public engagement, advocacy, network development, partnership building, and the implementation of intervention strategies to consider (Pumar-Méndez et al. 2022; Sharma 2021), most notably digital literacy and access to digital devices and platforms (Khafizova et al. 2023; Pang, Lee & Murshed 2023). Achieving these outcomes in a digital format requires careful and coordinated planning, as the design process can be complex and multifaceted (Abdel-Aziz et al. 2022; Houbby et al. 2020; Palumbo, Nicola & Adinolfi 2022).

Behaviour change is central to the uptake of health information by the public, and equally relevant to shifting the behaviours of educators and students towards embracing new modes of disease prevention and health promotion. The Theory of Planned Behaviour (TPB), developed by Ajzen (1991), offers a framework for health educators to understand the required shifts. The theory proposes that behavioural intention is shaped by attitudes towards the behaviour, perceived social norms, and perceived control over performing the behaviour. Within the context of our study, this behavioural intention predicts the likelihood of behavioural change required to implement digital health promotion and disease prevention strategies.

Health professions educators have a social responsibility to deliver healthcare professionals who can creatively provide information to promote health and address the healthcare needs of communities, while promoting behavioural change (Mahdavynia et al. 2022). While research on digital health communication examines the use and impact of specific technologies and applications (Lupton 2014; Shen et al. 2024), little is known about how undergraduate students in health professions education programmes create digital artefacts for health promotion and disease prevention. This article addresses this gap by describing research reporting on undergraduate students’ development of digital artefacts for health promotion and disease prevention in health professions education programmes. By exploring these approaches, our study offers educators practical insights into integrating digital health communication into curricula through the lens of the TBP, thereby aligning healthcare education with the evolving demands of modern healthcare delivery.

## Research methods and design

A scoping review guided the identification of current literature on digital artefacts developed for health promotion and disease prevention by undergraduate students in health professions education.

### Study design and search strategy

The scoping review protocol is registered on the Open Science Framework (osf.io/3yrmv) and reported according to the Preferred Reporting Items Extension for Scoping Reviews guidelines (PRISMA-ScR) as described by Aromataris et al. (eds. 2024). The review question ‘*What digital artefacts have been developed for health promotion and disease prevention by undergraduate students in health professions education programmes?*’ aligned with elements of the PCC (Population, Concept, Context) mnemonic (eds. Aromataris et al. 2024; Peters et al. 2020). The search strategy included the following core concepts: ‘Undergraduate healthcare students’, ‘digital artefacts’ and ‘Health promotion and/or disease prevention’. Table 1 presents the formulation of the search strategy, which was executed with the assistance of a senior librarian at a university, experienced in review-based research. The proposed nine-step research approach framework to systematically search the literature and select appropriate records was used (Peters et al. 2021). No date or language limiters were set during the search to ensure a robust description of all the relevant available literature.

**TABLE 1:** Search terms and synonyms used to facilitate the collection of literature.

Criteria	Keyword and/or phrase
Population	((student* or undergraduate* or Baccalaur*) n3 (“Health* Occupation*” or “Allied Health*” or “health* profession*” or “health* science*” or nurs* or medical or medicine or physiotherap* or “physical therap*” or pharma* or “occupational therap*” or optometr* or “speech therap*” or audiolog* or dietetic* or paramedic* or dentis* or dental or biokinetic*))
Concept	(((electronic or Digit*) n2 (content* or artifact* or artefact* or modalit* or technol* or modalit* or means or strategy* or method or methods or product* or pamphlet* or story* or stories)) or video or videos or podcast* or storyboard* or audio* or youtube or tiktok or instagram* or whatsapp or “socal media*” or canva or “visual aid*”)
Context	((patient* or Health*) n3 (promot* or inform* or educat*)) or (illness* or disease*) n2 prevent*))

A search of 13 electronic databases was performed in collaboration with the librarian in August 2025. Databases included Academic Search Ultimate, Africa-Wide Information, APA PsycArticles, APA PsycInfo, Applied Science & Technology Source Ultimate, CAB Abstracts with Full Text, CINAHL with Full Text, Communication & Mass Media Complete, ERIC, GreenFILE, Health Source: Nursing/Academic Edition, MEDLINE and Sociology Source Ultimate.

### Selection criteria

Published peer-reviewed records describing digital artefacts created by undergraduate health professions students in health professions education programmes were included. Unrelated records, grey literature not presenting a detailed description of student-created digital artefacts, duplicates, records exploring the use of technology in teaching and learning and records to which the authors could not gain full-text access were excluded. Table 2 depicts the inclusion and exclusion criteria used by the authors.

**TABLE 2:** Inclusion and exclusion criteria.

Criteria	Inclusion	Exclusion
Language	Studies in all languages will be retrieved. If an article is not available in English, the authors will request an English copy from the author(s).	Studies in other languages for which English versions are not available
Type of literature	Primary studies are accessible through the UFS library	Studies which are inaccessible through the library or other means available to the authors
Sources	Published peer-reviewed articles	Secondary studies, for example, reviews and grey literature
Population and sample	All undergraduate students in HPE	Postgraduate students and/or other professionals

UFS, University of the Free State; HPE, health professions education.

The first author contacted the corresponding authors of records in languages other than English for English versions. If English version records could not be traced, they were excluded. Reference lists of included records were also reviewed to identify additional records for inclusion.

### Data collection

**FIGURE 1:**
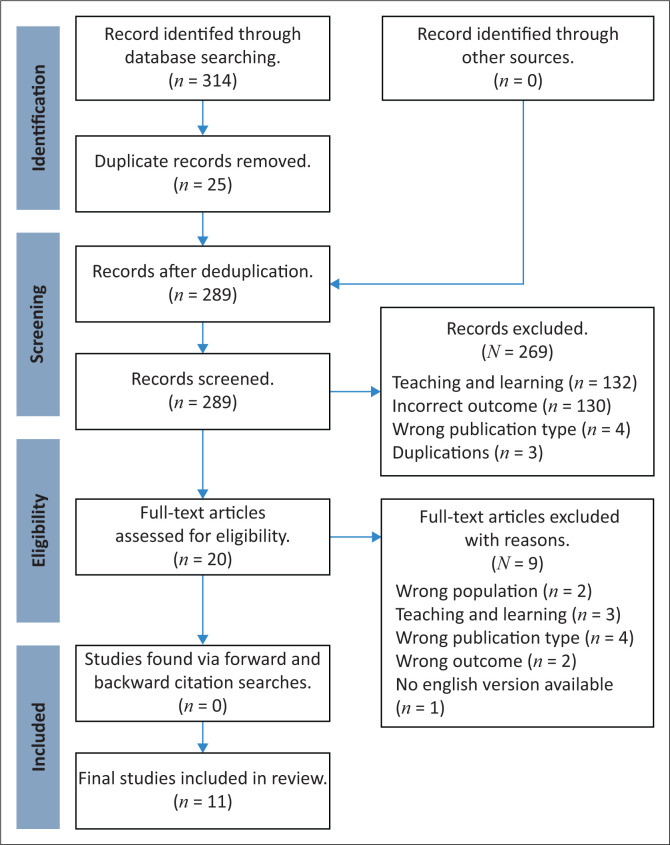
Preferred Reporting Items Extension for Scoping Reviews (PRISMA-ScR) flow diagram for scoping reviews.

A total of 314 records were identified for possible inclusion and were uploaded onto Rayyan, an online review platform, to facilitate the review. After automatic de-duplication, 289 titles and abstracts were independently screened by the two authors based on the inclusion and exclusion criteria. Twenty full-text articles were identified following the further exclusion of 269 records with reasons provided (Figure 1).

From the full-text articles, nine articles were determined to be irrelevant to the topic at hand through independent author screening and were excluded. In addition, an ancestral search was performed on the full-text articles included for possible inclusion; however, none were found. Discrepancies were resolved by discussing the outcome of each screening round to promote the rigour of selected articles. No mediation was required. A total of eleven articles were included in the final analysis, as depicted in the PRISMA flow diagram in Figure 1. Selected articles were appraised according to the Critical Appraisal Checklist for educational intervention (Morrison et al. 1999). The appraisal performed for our study was not for inclusion and exclusion purposes but rather for discussion.

### Data extraction

The authors independently extracted relevant data from the included articles using an author-developed data extraction sheet. The following data were extracted: citation; publication year; country; study design; student population; format, content, and method of sharing the digital artefact; student preparation; the topic and population of the digital artefact aimed at; and study outcomes. Each author subsequently reviewed the other author’s extraction data to verify accuracy and strengthen methodological rigour. No discrepancies in the data extracted following independent data checking were observed.

### Data analysis

Data were extracted literatim in an Excel spreadsheet (Online Appendix 1 Table 1-A1). Thereafter, the data were analysed by the primary author under our study and content characteristics. Our study’s characteristics were reported as quantitative data using graphs and figures to illustrate findings. The content characteristics were inductively thematically analysed and displayed in tables and described narratively. Identified themes were verified by the second author to ensure alignment with the analysed data, and consensus was reached through discussion between both authors.

### Ethical considerations

Ethical clearance to conduct our study was obtained from the Health Sciences Research Ethics Committee at the University of the Free State (reference number: UFS-HSD2024/0153).

## Results

### Study’s characteristics

From the 11 studies, no studies were identified prior to 2014, with most of the studies (*n* = 3) being published in 2018 (Amherdt, Kim & Basir 2018; Gawlink, Jeu & Reisinger 2018; Unterseher 2018), as shown in Table 3.

**TABLE 3:** Summary of articles included.

Authors	Published	Study methodology	Quality appraisal score (%)	Country	Student population	Study aim
Abdel-Aziz et al.	2022	Quantitative	78	Egypt	Medicine	To train university students to design a video-based health education programme for promoting peer-to-peer education and community awareness
Amherdt et al.	2018	Quantitative	89	United States	Medicine	To test educational modules developed by a medical student without extensive multimedia production training
Drake et al.	2017	Quantitative	67	United States	Nursing	To create an educational campaign to bolster seat belt use
Fliorent et al.	2023	Quantitative	67	United States	Medicine	To create South Jersey Skin Talk (SJST) to improve dermatologic health literacy in skin-of-colour communities, particularly in underserved areas
Gawlink et al.	2018	Quantitative	89	United States	Nursing	To spread awareness on mental illness, give the general public the skills and resources needed to identify and assist individuals who are struggling with mental illness and decrease the stigma surrounding mental illness
Houbby et al.	2020	Not reported	44	United Kingdom	Medicine	To reduce the stigma around mental health in the Arab patient population through a stop-motion video
Mangadu	2014	Not reported	67	United States	Health Promotion	To create digital stories to address peer health issues
Masters	2014	Quantitative	78	Oman	Medicine and Laboratory	To teach medical students to develop their own medical apps
Piscitelli et al.	2020	Not reported	56	Italy	Nursing, Medicine and other health professions	To describe a novel approach to promote the creation of innovative educational tools to improve knowledge of and compliance with hand hygiene rules among healthcare and medical students
Ramón-Arbués	2025	Mixed methods	78	Spain	Nursing	To assess student satisfaction, perceptions and learning experience regarding the utility of a teaching activity centred on creating health education videos
Unterseher	2018	Mixed methods	67	United States	Nursing	To create a social marketing campaign based on a topic area from Healthy People 2020

Note: Please see full reference list of this article, Van der Merwe, A. & Hugo L., 2026, ‘Clinical education, digital artefacts, disease prevention, health promotion and undergraduate healthcare students’, *South African Journal of Physiotherapy* 82(1), a2298. https://doi.org/10.4102/sajp.v82i1.2298, for more information.

Most studies (*n* = 10) were performed in high-income countries, with the United States (*n* = 6) being the lead (Table 2). Only one (*n* = 1) country, Oman, was classified as a lower-middle-income country according to the World Bank Classifications (World Bank 2024). All studies except one (*n* = 1) were research articles with more than half (*n* = 6) employing purely quantitative methodology (Table 3). Medical students (*n* = 6) were involved in the creation of most of the artefacts, followed by nursing students (*n* = 5). Four studies (*n* = 4) included second-year students (Amherdt et al. 2018; Ramón-Arbués et al. 2025; Unterseher 2018; Drake et al. 2017), while five (*n* = 5) did not report the year level of students (Abdel-Aziz et al. 2022; Fliorent et al. 2023; Gawlink et al. 2018; Mangadu 2014; Masters 2014). The overall quality of studies as measured on the critical appraisal checklist for an article on an educational intervention (Morrison et al. 1999) was good, with most studies (*n* = 9) scoring above 65% (Table 3).

### Content characteristics

Six studies reported the creation of video-based materials (Abdel-Aziz et al. 2022; Amherdt et al. 2018; Houbby et al. 2020; Mangadu 2014; Ramón-Arbués et al. 2025; Unterseher 2018), while others used multimedia or online platforms (Drake et al. 2017; Gawlink et al. 2018; Piscitelli et al. 2020). All studies developed artefacts for health-specific topics across various populations, with some targeting multiple populations (Table 4).

**TABLE 4:** Target population included.

Target population included	Authors
Culture-specific	Fliorent et al. (2023), Houbby et al. (2020), Mangadu (2014)
Healthcare specific	Fliorent et al. (2023), Houbby et al. (2020), Piscitelli et al. (2020), Amherdt et al. (2018), Gawlink et al. (2018), Mangadu (2014)
Non-specified	Abdel-Aziz et al. (2022), Unterseher (2018), Ramón-Arbués et al. (2025)
School-specific	Piscitelli et al. (2020), Drake et al. (2017), Masters (2014)
Law enforcement	Gawlink et al. (2018)

Note: Please see full reference list of this article, Van der Merwe, A. & Hugo L., 2026, ‘Clinical education, digital artefacts, disease prevention, health promotion and undergraduate healthcare students’, *South African Journal of Physiotherapy* 82(1), a2298. https://doi.org/10.4102/sajp.v82i1.2298, for more information.

Findings showed that most artefacts were directed at a healthcare-specific population, followed by non-, cultural and school-specific population groups. Law enforcement was only reported in one study.

### Methods of sharing

Methods of sharing artefacts included using social media platforms such as Instagram and Pinterest (Abdel-Aziz et al. 2022; Drake et al. 2017; Fliorent et al. 2023; Gawlink et al. 2018; Ramón-Arbués et al. 2025; Unterseher 2018), followed by video screenings (Houbby et al. 2020; Mangadu 2014; Unterseher 2018). Ramón-Arbués et al. (2025), Gawlink et al. (2018) and Unterseher (2018) specifically note the usage of different platforms for sharing digital artefacts. However, not all created artefacts were shared. Amherdt et al. (2018) reported sharing their artefacts with their study population on an iPad within the controlled research setting, stating that the created artefacts had the potential to be shared via various online methods (Amherdt et al. 2018).

### Student’s preparation

Contextual support included the provision of expert input (Abdel-Aziz et al. 2022; Drake et al. 2017; Gawlink et al. 2018; Unterseher 2018), engagement with the target population (Amherdt et al. 2018; Houbby et al. 2020) and educator feedback (Abdel-Aziz et al. 2022; Mangadu 2014; Ramón-Arbués et al. 2025) to ensure information accuracy and relevance. Moreover, theoretical content (Abdel-Aziz et al. 2022; Drake et al. 2017; Ramón-Arbués et al. 2025), engagement in digital content-sharing workshops (Abdel-Aziz et al. 2022; Ramón-Arbués et al. 2025), access to technical support and resources, and completion of a medical informatics course (Masters 2014) aided in preparing and supporting students to fulfil the assigned tasks. Two articles did not mention any form of student preparation or support (Fliorent et al. 2023; Piscitelli et al. 2020).

### Outcomes

Table 5 displays emerging themes regarding the perceived outcomes of studies where digital artefact creation by students in undergraduate healthcare teaching and learning was undertaken.

**TABLE 5:** Outcomes of studies.

Theme	Sub-theme	Category
Increased engagement	Target population	Culturally relevant Increased knowledge Empowerment
	Students	Increased knowledge and skills Empowerment
Accessibility	-	Easy to create/use Various platforms Cost-effective
Messages	-	Resonate
Curriculum	-	Adapt Planning Instructional design Limited educational theory frameworks or models

Results of our study indicated benefits for both target populations and students. Target populations experienced empowerment (Fliorent et al. 2023; Houbby et al. 2020), increased knowledge (Abdel-Aziz et al. 2022; Amherdt et al. 2018; Fliorent et al. 2023), and perceived cultural relevance (Abdel-Aziz et al. 2022; Fliorent et al. 2023; Houbby et al. 2020). Students felt empowered (Abdel-Aziz et al. 2022; Houbby et al. 2020; Unterseher 2018) and reported increased content and digital skills (Abdel-Aziz et al. 2022; Houbby et al. 2020; Mangadu 2014; Ramón-Arbués et al. 2025; Unterseher 2018). Digital artefacts were accessible through ease of creation and use (Amherdt et al. 2018; Fliorent et al. 2023; Houbby et al. 2020; Unterseher 2018), distributable across platforms (Abdel-Aziz et al. 2022; Amherdt et al. 2018; Piscitelli et al. 2020; Ramón-Arbués et al. 2025; Unterseher 2018), and cost-effective (Amherdt et al. 2018; Houbby et al. 2020; Mangadu 2014).

Additionally, recommended positive messaging should resonate with the intended audience (Drake et al. 2017), be informative, emotive and entertaining (Drake et al. 2017; Fliorent et al. 2023; Piscitelli et al. 2020; Unterseher 2018). The final theme highlighted curriculum adaptation needs namely integrating digital artefact development (Houbby et al. 2020; Masters 2014; Ramón-Arbués et al. 2025), intentional planning (Abdel-Aziz et al. 2022; Gawlink et al. 2018; Mangadu 2014; Ramón-Arbués et al. 2025), and applying sound instructional design principles for digital skills development (Abdel-Aziz et al. 2022; Drake et al. 2017; Gawlink et al. 2018; Ramón-Arbués et al. 2025). Only two studies reported using an underpinning educational theory, framework or model (Mangadu 2014; Ramón-Arbués et al. 2025).

## Discussion

This scoping review aimed to describe undergraduate students’ development of digital artefacts for health promotion and disease prevention in health professions education programmes. Health behaviours are central to effective health promotion and disease prevention, with digital technology increasingly embedded within healthcare (Pang et al. 2023). Looking at our study characteristics, despite the widespread uptake of mobile technology globally (Erku et al. 2023; Kruk et al. 2018) and the critical role of healthcare training institutions in preparing world-ready graduates (Del Riccio et al. 2023; Stellefson et al. 2020), it is concerning that only 11 articles were eligible for this review. This limited number suggests that educators might be focused on implementing student-produced videos as part of day-to-day teaching and learning and potentially not harnessing their power for the purpose of health promotion and disease prevention within a changing clinical space (Ramón-Arbués et al. 2025).

With most studies originating from high-income countries, this imbalance reflects an under-representation of low-middle-income contexts, despite their unique digital access challenges (Yardley et al. 2016). As reported by STATS SA, 78.6% of South African households had some form of internet access in 2023 (Independent Communications Authority of South Africa [ICASA] 2025). However, only 14.5% had internet access at home, likely reflecting the continued reliance on mobile internet as the primary means of connectivity (ICASA 2025). With increased connectivity, even in rural areas, the potential benefits of utilising digital means for health promotion have increased significantly (Koh et al. 2021). Although digital access has increased, it is advised that developed digital artefacts should remain simple in design, low in data demand, and available both online and offline, while being tailored to the content, context, and population for which they are intended to increase their reach even to those who may be digitally illiterate initially (Koh et al. 2021; Yardley et al. 2016). Given that student-creators themselves may encounter challenges with resource access and digital literacy, emphasising the use of widely available software and personal devices is essential, as such approaches have been shown to support the development of professional-quality digital artefacts (Ramón-Arbués et al. 2025).

The absence of the inclusion of rehabilitation students as creators of digital artefacts in the included studies is worrying. This omission is particularly significant for rehabilitation professionals, who play a critical role in health promotion and disease prevention through their expertise in functional recovery, lifestyle modification, and community-based interventions (WHO 2017). This identified gap necessitates investigations to ensure the rehabilitation professions can increase patient reach through targeted upskilling initiatives that equip them with the digital literacy and technological competencies essential for creating effective digital artefacts. Drawing on the TPB, the findings of our study identify essential considerations for guiding healthcare educators in facilitating students in the creation of effective digital artefacts for health promotion and disease prevention, which may ultimately lead to changed behaviour of communities. The content results will be discussed at the hands of the three core components of the TPB, namely, attitudes, subjective norms, and perceived behavioural control.

### Attitudes

Within the TPB framework, attitudes towards digital artefact creation are shaped by perceived outcomes and benefits (Asare 2015). Records included in our study reported dual benefits for both creator and recipient knowledge, strengthening positive attitudes towards health behaviour and digital artefact creation by providing immediate, tangible rewards (Asare 2015; Panahi et al. 2022; Unterseher 2018). If implemented correctly, healthcare students’ attitudes towards creating digital artefacts may be positively influenced by their feelings of empowerment in sharing healthcare messages in an alternative and impactful format (Drake et al. 2017; Gawlink et al. 2018; Unterseher 2018). Students in included studies reported increased knowledge and skills, suggesting digital artefact creation focusing on health promotion and disease prevention facilitates active learning (Ramón-Arbués et al. 2025; Unterseher 2018; Vallentin-Holbech et al. 2020). This outcome can be credited to the engagement of content experts, improved subject knowledge, and ensured accuracy, limiting misinformation as highlighted by several authors (Abdel-Aziz et al. 2022; Drake et al. 2017; Gawlink et al. 2018; Unterseher 2018). Such first-hand experiences can positively shape students’ attitudes towards this challenging task, enhancing their appreciation of its impact and strengthening their confidence in their ability to create meaningful and effective products (Houbby et al. 2020). In addition, recipients from target populations reported learning benefits (Abdel-Aziz et al. 2022; Amherdt et al. 2018; Fliorent et al. 2023), empowerment and greater satisfaction when digital artefacts were perceived as culturally appropriate and meaningful (Abdel-Aziz et al., 2022; Fliorent et al. 2023; Houbby et al. 2020).

To enhance the benefits for both students and recipients, healthcare curricula should increase access to health information through various digital means (Khafizova et al. 2023; Pang et al. 2023). The physical accessibility of digital content can be enhanced through sharing information in a variety of formats (Gawlink et al. 2018; Unterseher 2018) across platforms, ranging from social media (Abdel-Aziz et al. 2022; Fliorent et al. 2023; Gawlink et al. 2018; Piscitelli et al. 2020; Ramón-Arbués et al., 2025; Unterseher 2018), video screenings (Houbby et al. 2020; Mangadu 2014) and online forums, such as websites or YouTube (Amherdt et al. 2018; Gawlink et al. 2018). In our study, results indicated that social media was mostly used when developing and sharing digital artefacts. Globally, social media has been integrated into health interventions to reach a large and geographically dispersed population pool (Koh et al. 2021) to provide access to health resources while motivating health behaviour change (Chen & Wang 2021; Drake et al. 2017; Fliorent et al. 2023). Video-based materials are also a frequent format of digital artefact and allow for the creation of inclusive content by including diverse visuals and various languages (Amherdt et al. 2018; Houbby et al. 2020). A positive link towards users’ adoption and continued use of short videos as a source of health information has been reported (Song et al. 2021) as it increases accessibility and limits data required if the content could easily be downloaded and accessed offline (Koh et al. 2021; Song et al. 2021), which enhances positive attitudes towards digital artefact creation and use.

In addition, we found that how the health promotion and prevention messages are communicated is vital. The communication in health messages should be jargon-free, but remains a challenge (Yardley et al. 2016), especially when sharing information in an asynchronous format. Educators should train students to create brief, easily understood, informative, entertaining, and emotive content (Drake et al. 2017; Houbby et al. 2020; Yardley et al. 2016). This review highlighted that a positive communicative approach to message content is preferred (Drake et al., 2017) as people are more inclined to share encouraging and hopeful health messages (Chang et al., 2022). Although fear-based messages might induce motivation to improve lifestyles, positive messaging has been reported to enhance information processing, strengthen perceptions of efficacy and contribute to positive behavioural outcomes (Ort, Siegenthaler & Fahr 2021). The complexity of curating impactful content is therefore highlighted, and it is advised that educators spend time with their students in developing this essential, albeit difficult skill.

The study’s findings revealed that digital artefact creation was perceived as cost-effective, with students preferring to use readily available resources (Ramón-Arbués et al. 2025). A cost-effective approach can decrease resource barriers, negatively influencing attitudes. The ability to distribute content across various platforms also enhances the perceived value proposition for both students and target populations (Amherdt et al. 2018). The importance of selecting user-friendly platforms, developing digital literacy and providing adequate technical support to maintain positive attitudes towards digital health communication tasks is highlighted (Ramón-Arbués et al. 2025; Unterseher 2018). This practical advantage contributes to more positive attitudes by reducing resource-related concerns that could otherwise deter student engagement.

### Subjective norms

The subjective norms of the TPB signify the social aspect of behaviour, highlighting the influence of social expectations and perceptions on individual choices (Asare 2015). The findings revealed that subjective norms were significantly shaped by institutional support structures and professional expectations within healthcare education. Expert input during the digital artefact creation process emerged as a critical social influence, with students perceiving this guidance as both an expectation and enabler of quality content development (Abdel-Aziz et al. 2022; Drake et al. 2017; Gawlink et al. 2018; Unterseher 2018). Similarly, educator feedback (Abdel-Aziz et al. 2022; Ramón-Arbués et al. 2025; Mangadu 2014) served as a normative influence, emphasising ethical standards for content accuracy and relevance (Fliorent et al. 2023; Koh et al. 2021). This institutional support creates an educational environment where digital artefact creation is viewed as professionally endorsed and academically valued.

The role of direct engagement with the target populations in shaping subjective norms was also highlighted in included studies. Social accountability and the perceived importance of their work are reinforced when students interact with their recipients during the development process (Houbby et al. 2020; Unterseher 2018). Positive feedback from target populations served as powerful social validation (Fliorent et al. 2023; Houbby et al. 2020), reinforcing students’ commitment to creating culturally appropriate and impactful content.

Digital artefacts for health promotion and disease prevention can target individual, organisational, community and population levels. However, considering the link between users, digital artefacts and the socio-cultural environment (Abdel-Aziz et al. 2022; Fliorent et al. 2023; Yardley et al. 2016), targeting several levels simultaneously is most effective (Laverack 2017; Patey et al. 2023). The predominant use of social media platforms for sharing digital artefacts in our study also reflects broader societal norms about information sharing and communication. Scoping review results indicated that, depending on the healthcare topic, single or multiple populations may be targeted across multiple platforms when designing artefacts, thereby addressing the global drive for increased health promotion reach (Koh et al. 2021). As healthcare systems increasingly recognise the need for accessible, culturally sensitive health information (Houbby et al. 2020; Koh et al. 2021), it is recommended that students engage with digital artefact creation as part of professional development during their training.

The convergence of institutional support, community engagement, and professional expectations creates powerful subjective norms encouraging students to view digital artefact creation as a legitimate and important aspect of their healthcare education (Houbby et al. 2020). These social influences collectively create an environment where students perceive digital artefact creation as both expected and valued (Unterseher 2018), contributing to stronger intentions to engage in this educational activity.

### Perceived behavioural control

Within the TPB, perceived behavioural control refers to a person believing they will be capable of changing the behaviour (Asare 2015). With technology interwoven into the fabric of society and educational settings, it is not surprising that students were able to, and enjoyed creating, digital artefacts even with limited technological support (Amherdt et al. 2018; Gawlink et al. 2018; Mangadu 2014; Unterseher 2018). The improved technological skills of twenty-first-century students and their access to basic digital devices enable them to create cost-effective digital artefacts to promote healthcare and prevent disease (Ramón-Arbués et al. 2025). As the current generation enrolled in tertiary education institutions is adept at using basic technologies and social media (Ramón-Arbués et al. 2025), interestingly, most included studies did not require additional time to teach students technical skills for artefact creation, which may highlight a gap in healthcare curricula. While technical training was minimal across studies, with only one study observing scheduled technology training for more complex assignments such as basic healthcare applications (Masters 2014), time was allocated in several studies to assist students in creating impactful and accurate content (Abdel-Aziz et al. 2022; Gawlink et al. 2018; Mangadu 2014; Masters 2014). This approach, in alignment with the TPB, is essential to foster students’ confidence in their ability to create meaningful and impactful content while ensuring information accuracy and successful task completion.

Adapting healthcare curricula to ensure student proficiency in integrating applicable and sustainable technologies into patient management is vital (Khafizova et al. 2023; Pang et al. 2023; Ramón-Arbués et al. 2025). While the results demonstrate positive outcomes, the limited reported educational frameworks or models underpinning the educational approaches may impact educators’ confidence and ability to implement digital artefact creation effectively. This is concerning, as educational theory should underpin all considerations when developing learning and teaching activities to ensure optimal student engagement and motivation for both learning and societal change (eds. El Hakim & Lowe 2020). Limitations of our study include the small sample retrieved from the sources searched.

## Conclusion

Through this scoping review, we aimed to describe undergraduate students’ development of digital artefacts for health promotion and disease prevention in health professions education programmes. Our study highlighted both the potential and gaps in undergraduate healthcare students creating digital artefacts for health promotion. While findings show positive outcomes for student creators and target populations, limited studies available suggest underutilisation of this educational approach. The predominance of medical and nursing students, with minimal rehabilitation professional involvement, indicates missed opportunities for diverse healthcare perspectives. Applying the TPB highlights critical success factors. Students’ positive attitudes were fostered through perceived benefits, including knowledge enhancement and culturally relevant content creation. Subjective norms were shaped by institutional support and expert guidance, while perceived behavioural control was enhanced by technological competencies and appropriate support structures. However, most studies lacked underlying educational frameworks, representing a significant implementation challenge. Information from our study can guide educators in health professions training programmes to assist students in the development of digital artefacts for health promotion and disease prevention to address healthcare needs. It is recommended that healthcare educators move beyond ad hoc approaches to embrace theoretically grounded pedagogical strategies that systematically integrate digital health promotion into curricula.
